# Computed Tomography Pulmonary Angiography Prediction of Adverse Long-Term Outcomes in Chronic Thromboembolic Pulmonary Hypertension: Correlation with Hemodynamic Measurements Pre- and Post-Pulmonary Endarterectomy

**DOI:** 10.3390/tomography9050142

**Published:** 2023-09-26

**Authors:** Deepa Gopalan, Jan Y. J. Riley, Kai’en Leong, Senan Alsanjari, William Auger, Peter Lindholm

**Affiliations:** 1 Department of Physiology and Pharmacology, Karolinska Institute, 171 77 Stockholm, Sweden; peter.lindholm@ki.se; 2 Department of Radiology, Imperial College Hospital NHS Trust, London W12 0HS, UK; senan.alsanjari4@nhs.net; 3 Department of Diagnostic Imaging, Monash Health, Melbourne 3168, Australia; jyjriley@gmail.com; 4 Department of Cardiology, Royal Melbourne Hospital, Melbourne 3052, Australia; leongkaien@gmail.com; 5 Department of Pulmonary Medicine, University of California, San Diego, CA 92037, USA; williamrauger@icloud.com; 6 Department of Emergency Medicine, University of California, San Diego, CA 92103, USA

**Keywords:** CTPA planimetry, CTEPH, pulmonary endarterectomy

## Abstract

CT pulmonary angiography is commonly used in diagnosing chronic thromboembolic pulmonary hypertension (CTEPH). This work was conducted to determine if cardiac chamber size on CTPA may also be useful for predicting the outcome of CTEPH treatment. A retrospective analysis of paired CTPA and right heart hemodynamics in 33 consecutive CTEPH cases before and after pulmonary thromboendarterectomy (PTE) was performed. Semiautomated and manual CT biatrial and biventricular size quantifications were correlated with mean pulmonary artery pressure (mPAP), pulmonary vascular resistance (PVR) and cardiac output. The baseline indexed right atrioventricular volumes were twice the left atrioventricular volumes, with significant (*p* < 0.001) augmentation of left heart filling following PTE. Except for the left atrial volume to cardiac index, all other chamber ratios significantly correlated with hemodynamics. Left to right ventricular ratio cut point <0.82 has high sensitivity (91% and 97%) and specificity (88% and 85%) for identifying significant elevations of mPAP and PVR, respectively (AUC 0.90 and 0.95), outperforming atrial ratios (sensitivity 78% and 79%, specificity 82% and 92%, and AUC 0.86 and 0.91). Manual LV:RV basal dimension ratio correlates strongly with semiautomated volume ratio (r 0.77, 95% CI 0.64–0.85) and is an expeditious alternative with comparable prognostic utility (AUC 0.90 and 0.95). LV:RV dimension ratio of <1.03 and ≤0.99 (alternatively expressed as RV:LV ratio of >0.97 and ≥1.01) is a simple metric that can be used for CTEPH outcome prediction.

## 1. Introduction

Chronic thromboembolic pulmonary hypertension (CTEPH) is a form of pulmonary hypertension (PH) caused by pulmonary vasculature obstruction due to an organized thrombus, leading to an increase in right ventricular (RV) afterload and eventually RV failure. Pulmonary thromboendarterectomy (PTE) is the treatment of choice in eligible patients as it provides excellent symptomatic and prognostic improvement [1]. Within 2–3 weeks of successful surgery, there is rapid reverse RV remodeling with improvement in RV volumes and function [2]. However, surgery is not without risks, and the presence of a dilated RV with low systolic function and pulmonary vascular resistance (PVR) >600 dynes/s/cm^2^ are strong predictors of poor prognosis [2]. Despite continuing improvements in the safety and efficacy of PEA, with a perioperative mortality rate of <5% in expert CTEPH centers [3,4], there are specific complications such as residual PH and reperfusion lung injury that increase mortality and morbidity following surgery. Up to 50% of patients may have persistent PH after surgery, which can affect long-term outcomes [5]. Post-surgical PH has been shown to be associated with poorer functional capacity and increased mortality [6]. The largest prospective cohort study to date [7] showed that mean pulmonary arterial pressure (mPAP) ≥38 mm Hg and pulmonary vascular resistance (PVR) ≥5.3 WU after PTE correlated with a higher risk of death because of CTEPH.

While the correlation between the extent of technically accessible disease and PH severity is an important determinant for predicting hemodynamic improvement [3], post-surgical outcomes are also influenced by other factors such as concomitant cardiopulmonary disease and right heart failure [6]. Hence, a multiparametric assessment is needed to assess the risk/benefit ratio of surgery and its consequences. Non-ECG-gated CT pulmonary angiography (CTPA) is frequently used in CTEPH diagnosis as it allows for noninvasive detection of thrombi. CTPA can evaluate the location and extent of chronic thromboembolic changes and identify patients who are potentially suitable for PTE. CTPA images may also hold important prognostic information regarding the success of PTE, but this has not yet been investigated. 

The ventricular adaptations in CTEPH pathophysiology are well documented. Disease progression is characterized by worsening RV remodeling and RV dysfunction. Diastolic RV pressure increases in combination with low left ventricular (LV) preload and underfilling results in LV diastolic impairment [8]. The atrial changes are less well understood, although atrial compliance is an important determinant of cardiac performance. There is evolving data on magnetic resonance imaging emphasizing the importance of the right atrial (RA)–right ventricular axis in PH [9] and the contribution of RA function to maintenance of RV function in CTEPH [10]. Furthermore, there is an increasing trend to use atrial size and function as a morphophysiologic expression to predict cardiovascular mortality in left heart disease [11], but its importance in outcome prediction in CTEPH is unknown.

We undertook this study to determine if non-ECG-gated CTPA can be used to evaluate atrioventricular size in CTEPH and if the CT-derived chamber quantification before and after pulmonary thromboendarterectomy (PTE) can help identify patients with significant residual PH and thereby provide a noninvasive measure for long-term outcome prediction. 

## 2. Materials and Methods

### 2.1. Patient Population 

This retrospective study was approved by the Institutional Review Board (IRAS project ID 280472), and the need for informed consent was waived. The main aim of our study was to evaluate the biatrial and biventricular chamber sizes using CTPA before and after PTE. Our standard departmental policy is to perform CTPA and right heart catheterization (RHC) in all CTEPH patients prior to and 3–4 months following PTE. Of the 53 consecutive adult (>18 years) CTEPH patients who had undergone PTE between Jan 2014 and 2019, complete paired datasets (local CTPA and RHC, pre- and post-surgery) were available for 44 cases. Following a consensus decision, 11 patients were excluded due to suboptimal CT image quality (5 poor left atrial opacification, 6 significant cardio-respiratory motion artifacts), and 33 paired cases were included in the final evaluation. Clinical and laboratory data, including body surface area, atrial fibrillation, WHO functional class, renal function and BNP, were routinely assessed and extracted via the electronic medical record. Echocardiographic evidence of tricuspid regurgitation and its severity was recorded. 

### 2.2. CT Acquisition

Images were acquired on a 128-slice configuration scanner (Somatom Definition AS+; Siemens AG, Forcheim, Germany) using our routine non-electrocardiographic-gated protocol with the following standard acquisition parameters: 0.8 pitch, 0.6 mm collimation, table speed 61.4 mm/rotation, 0.5 s rotation time, tube current 100 mAs and tube voltage 80–120 kVp. Scanning was performed in a craniocaudal direction from the thoracic inlet to the diaphragm following injection of 70–100 mL Omnipaque 350 (GE Healthcare, Princeton, NJ, USA) at rates of 5 mL/s. Bolus tracking technique was performed with CARE bolus software (Siemens Medical Solutions, Forcheim, Germany), with the region of interest placed on the main pulmonary trunk and the trigger threshold set at 130 HU. Sequential axial images were reconstructed at a 1-mm slice thickness at a 1 mm interval using SAFIRE (Sinogram Affirmated Iterative Reconstruction, strength 3) iterative reconstruction and a standard 512 × 512 matrix size.

The time interval between CTPA and RHC pre-PTE varied between 1 and 19 days (median 1 day), while post-PTE, the 2 procedures happened either on the same day or within a 1-day interval. 

### 2.3. CT Measurement

The CT cardiac chamber quantification was performed in accordance with the well-established international recommendations in echocardiography [12,13] by two radiologists: R1, a cardiovascular radiologist with 15 years’ experience, and R2, a radiology imaging fellow, blinded to clinical outcome.

**Figure 1:**
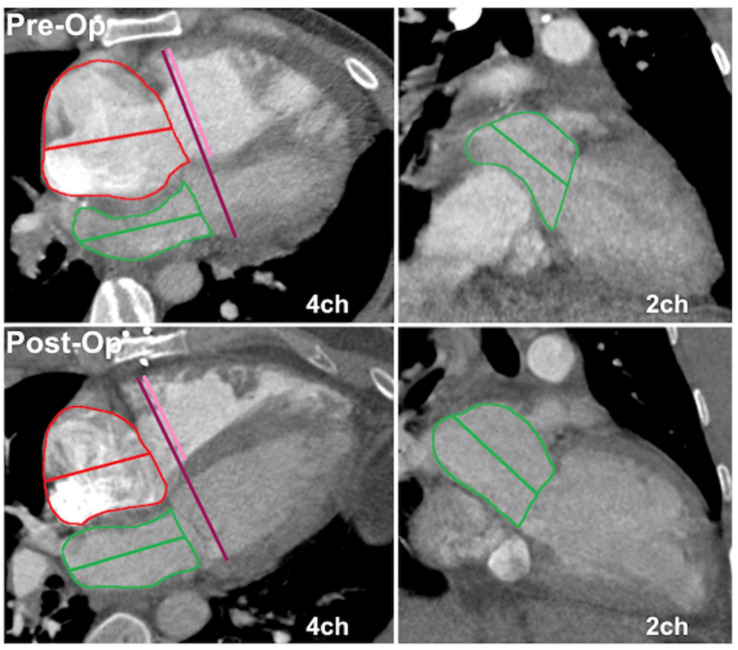
Atrial volumes and ventricular dimension ratio measurements on non-ECG-gated CTPA in a CTEPH patient pre- and post-endarterectomy. Top panels depict preoperative 4- and 2-chamber planes with corresponding postoperative imaging in the bottom row. Atrial areas planimetered with exclusion of appendages and pulmonary veins (left atrium). Atrial volumes calculated using 4 and 2-chamber areas and long axis diameter by area–length method. Total biventricular diameter measured from 4-chamber plane (**left panel**), extending from widest basal portion of RV compacted myocardium to LV endocardium. Interventricular septal thickness assigned to LV, with 4Ch basal LV diameter given by difference between total biventricular diameter and 4Ch basal RV diameter.

Biatrial and biventricular volumes were measured by semiautomated volume segmentation using a commercially available software (CVI42 version 5.12.1, Circle Cardiovascular Imaging, Calgary, AB, Canada). As specific postprocessing software might not be readily available to all CT readers, we also did simpler manual measurements of atrial volumes and the ventricular basal dimension ratio. Manual contouring for atrial volumes was performed using the Vitrea Advanced Visualization multimodal platform (Vital Images, Inc.; Minnetonka, MN, USA). Biventricular length was measured from the 4-chamber plane, extending from the widest basal portion of the RV to the LV endocardium (Figure 1). The ventricular basal dimension ratio was calculated as 4Ch basal LV diameter divided by basal RV diameter. 

All length measurements were recorded in centimeters (cm) and all area measurements in centimeters squared (cm^2^). The calculated volumes were indexed to body surface area. The time taken for loading and processing by the automated system and manual measurements was recorded. A detailed description of the methodology used for deriving the CT metrics is outlined in Appendix A. 

### 2.4. Right Heart Catheterization 

Patients were allowed only oral clear fluids prior to catheterization. A Swan–Ganz catheter was used for all pressure measurements. Pulmonary arterial wedge pressure (PAWP) tracings were obtained at end-expiration (mean of 3 tracings in sinus rhythm or 5 in AF). Cardiac output (CO) measurements were made using the thermodilution or Fick method. PVR (Wood units/WU) was calculated by (mPAP − mPAWP) ÷ CO. The various CT metrics were correlated with thresholds of mPAP ≥ 38 mm Hg and PVR ≥ 5.3 WU for adverse outcome prediction, as these hemodynamic values had been shown to be predictive of death [3].

### 2.5. Statistical Methodology 

Continuous normal data are presented as the mean (±1 standard deviation). Non-normal data are presented as the median (interquartile range). Continuous data were compared with a two-tailed Student’s *t*-test or Mann–Whitney U test for normal and non-normal data, respectively. Categorical variables are displayed as *n* (%), and differences were assessed using Fisher’s exact test. Intraclass correlation coefficient (ICC) estimates and their 95% confidence intervals for inter- and intra-observer variability in CT measurements were calculated. Correlation analysis was performed using the Pearson correlation coefficient or the Spearman’s rank test where the data were non-normal. Univariate analysis and multivariate stepwise logistic regression were used to identify atrial and ventricular size metrics that predicted elevated mPAP and PVR. Receiver operating characteristic (ROC) curves with area under the curve (AUC) values were used to compare the predictive ability of the various size metrics/ratios, and the optimal cut-points were defined by the Youden index. A *p*-value of ≤0.05 was considered statistically significant.

Paired pre- and post-operative catheterization data were used for evaluation of the various CT measures in the prediction of adverse hemodynamics. This was with the assumption that the relationship between static atrial/ventricular geometry and hemodynamics should remain reasonably constant regardless of operative status.

## 3. Results

The baseline characteristics are outlined in Supplementary Table S1. There were 18 men and 15 women, with a median age of 56 years. Before surgery, 94% were in WHO functional class III/IV with increased BNP. Following surgery, 70% were in WHO functional class I/II with a pronounced drop in BNP (*p* < 0.001). Preoperatively, 55% of cases had mild, and the remainder had moderate or severe tricuspid regurgitation (TR). Post-PTE, there was significant improvement, with 94% demonstrating either complete resolution or only mild residual regurgitation. No patient had tricuspid ring annuloplasty or valve replacement at the time of PTE. 

### 3.1. CT Semiautomated Volumes and Hemodynamics Pre- and Post-PTE: (Table 1 and Table 2) 

Preoperatively, the indexed RA volumes were more than twice the LA volumes, and the indexed RV volumes were twice the LV volumes. Following surgery, there was a significant augmentation of left heart filling, resulting in an increase in LA and LV volumes and a concomitant fall in the RA and RV volumes (*p* < 0.001). Significant improvements in hemodynamics were observed following surgery; mean pulmonary artery pressure (mPAP) fell from 45 ± 11 mmHg to 27 ± 12 mmHg (*p* < 0.001), and pulmonary vascular resistance (PVR) dropped from 9.0 ± 4.2 WU to 3.1 ± 1.9 WU (*p* < 0.001) with the increase in cardiac output (mean cardiac index (CI) 2.1 ± 0.7 to 2.6 ± 0.7; *p* < 0.001). The intraclass correlation coefficient (ICC 95% CI) for the 2 readers for CT chamber measurements was 0.99 (0.96–1.00) and 0.96 (0.76–0.99) for atria and ventricles, respectively. 

**Table 1:** CT volumes before and after pulmonary endarterectomy.

	Pre-op (*n* = 33)	Post-op (*n* = 33)	*p*-Value
Interval between CT and catheterization (days)	2 (1–22)	1 (1–1)	
LAVi (mL/m^2^)	25 ± 12	32 ± 10	<0.001
RAVi (mL/m^2^)	62 (45–89)	41 (31–54)	<0.001
LA:RA volume ratio	0.45 ± 0.27	0.76 ± 0.31	<0.001
LV volume indexed (mL/m^2^)	38 ± 11	51 ± 12	<0.001
RV volume indexed (mL/m^2^)	77 ± 31	51 ± 22	<0.001
LV:RV volume ratio	0.57 ± 0.29	1.09 ± 0.30	<0.001

LAVi: left atrial volume indexed; RAVi: right atrial volume indexed; LV: left ventricle; RV: right ventricle.

**Table 2:** Right heart catheterization-derived hemodynamics before and after pulmonary endarterectomy.

	Pre-op (*n* = 33)	Post-op (*n* = 33)	*p*-Value
PA systolic (mmHg)	77 (69–86)	40 (31–50)	<0.001
PA diastolic (mmHg)	27 ± 9	17 ± 8	<0.001
mPAP (mmHg)	45 ± 11	27 ± 12	<0.001
PVR (Wood units)	9.0 ± 4.2	3.1 ± 1.9	<0.001
mRAP (mmHg)	11 ± 5	7 ± 4	<0.001
mPAWP (mmHg)	12 ± 4	11 ± 4	NS
CO (L/min)	4.2 ± 1.7	5.4 ± 1.5	0.001
CI (L/min/m^2^)	2.1 ± 0.7	2.6 ± 0.7	<0.001

PA: pulmonary artery; mPAP: mean pulmonary artery pressure; mPAWP: mean pulmonary artery wedge pressure; mRAP: mean right atrial pressure; PVR: pulmonary vascular resistance; NS: Not Significant.

### 3.2. Correlation between CT Metrics and Hemodynamics: (Table 3)

**Figure 2:**
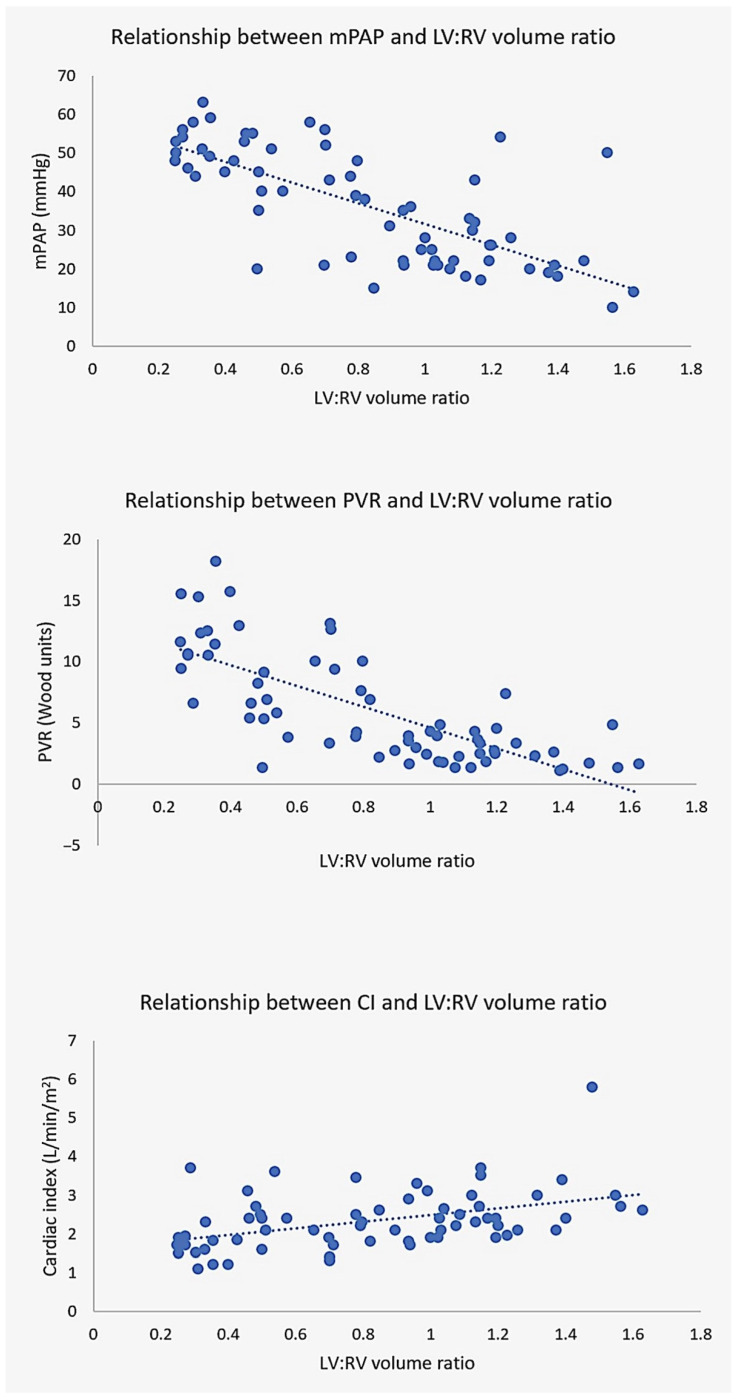
Relationship between LV:RV volume ratio and hemodynamics. CT ventricular volume ratio demonstrates excellent correlation with mean pulmonary artery pressure mPAP (**top**), pulmonary vascular resistance PVR (**middle**) and cardiac index CI (**bottom**).

CT volumetric ratios (atrial and ventricular) had stronger relationships with hemodynamic parameters than individual chamber measurements. Except for the indexed LA volume to cardiac index (LAVi–CI) relationship, all other chamber volumes and ratios were otherwise significantly correlated (all *p* ≤ 0.05) with invasive hemodynamics. Of all metrics, ventricular volume ratio was most closely related to mPAP, PVR and CI (Figure 2).

**Table 3:** Correlation between CT chamber quantification and invasive hemodynamics.

	mPAP	PVR	CI
LAVi	−0.36 (−0.55 to −0.13) *p* = 0.003	−0.43 (−0.61 to −0.21) *p* < 0.001	NS
RAVi	0.59 (0.41–0.73) *p* < 0.001	0.58 (0.39–0.72) *p* < 0.001	−0.38 (−0.57 to −0.15) *p* = 0.002
LA:RA volume ratio	−0.63 (−0.76 to −0.46) *p* < 0.001	−0.63 (−0.76 to −0.46) *p* < 0.001	0.38 (0.15 to 0.57) *p* = 0.002
LV volume indexed	−0.28 (−0.49 to −0.04) *p* = 0.023	−0.48 (−0.64 to −0.26) *p* < 0.001	0.36 (0.12–0.55) *p* = 0.003
RV volume indexed	0.72 (0.58–0.82) *p* < 0.001	0.68 (0.53–0.80) *p* < 0.001	−0.35 (−0.55 to −0.12) *p* = 0.004
LV:RV volume ratio	−0.72 (−0.82 to −0.58) *p* < 0.001	−0.75 (−0.84 to −0.62) *p* < 0.001	0.47 (0.25–0.64) *p* < 0.001

LAVi: left atrial volume indexed; RAVi: right atrial volume indexed; LV: left ventricle; RV: right ventricle; NS: Not Significant.

### 3.3. CT Prediction of Adverse Hemodynamics: (Table 4) 

Both atrial and ventricular ratios were able to predict prognostically adverse hemodynamics (mPAP ≥ 38 mmHg, PVR ≥ 5.3 WU), but the LV:RV volume ratio (sensitivity and specificity 91% and 88% and 97% and 86%, and AUC 0.90 and 0.95 for mPAP and PVR, respectively) outperformed the LA:RA volume ratio (sensitivity and specificity 78% and 82% and 79% and 92%, and AUC 0.86 and 0.91 for mPAP and PVR, respectively). A ventricular ratio cut point of ≤0.82 had a high sensitivity and specificity for identifying significant elevations of both mPAP and PVR.

The combination of atrial and ventricular measurements did not provide any additive effect, as of all the assessed atrial and ventricular ratios, stepwise logistic regression showed that only the LV:RV diameter ratio independently predicted adverse hemodynamics.

**Table 4:** CT prediction of adverse hemodynamics.

**CT for Prediction of mPAP > 38 mmHg**					
	**Cut Point for** **mPAP ≥ 38 mmHg**	**AUC of ROC** **(95% CI)**	***p*** **-Value**	**Sens.**	**Spec.**
LAVi (mL/m^2^)	≤29	0.73 (0.61–0.84)	<0.001	78%	65%
RAVi (mL/m^2^)	>43	0.79 (0.68–0.88)	<0.001	88%	68%
LA:RA volume ratio	≤0.59	0.86 (0.75–0.93)	<0.001	78%	82%
LV volume indexed (mL/m^2^)	≤37	0.67 (0.54–0.78)	0.016	53%	79%
RV volume indexed (mL/m^2^)	>54	0.92 (0.82–0.97)	<0.001	88%	88%
LV:RV volume ratio	≤0.82	0.90 (0.80–0.96)	<0.001	91%	88%
**CT for Prediction of PVR > 5.3 WU**					
	**Cut Point for** **PVR ≥ 5.3 WU**	**AUC of ROC** **(95% CI)**	***p*** **-Value**	**Sens.**	**Spec.**
LAVi (mL/m^2^)	≤29	0.75 (0.62–0.85)	<0.001	79%	62%
RAVi (mL/m^2^)	>44	0.84 (0.73–0.92)	<0.001	90%	68%
LA:RA volume ratio	≤0.47	0.91 (0.81–0.97)	<0.001	79%	92%
LV volume indexed (mL/m^2^)	≤42	0.76 (0.64–0.85)	<0.001	72%	70%
RV volume indexed (mL/m^2^)	>55	0.91 (0.82–0.97)	<0.001	90%	86%
LV:RV volume ratio	≤0.82	0.95 (0.86–0.99)	<0.001	97%	86%

LAVi: left atrial volume indexed; RAVi: right atrial volume indexed; LV: left ventricle; RV: right rentricle; PVR: pulmonary vascular resistance.

### 3.4. Comparison of CT Manual and Semiautomated Measurements

There was overall good agreement between manual and semiautomated measurement of biatrial volumes with LAV and RAV intraclass correlation coefficients (ICC 95% CI) at 0.84 (0.70–0.91) and 0.88 (0.80–0.92), respectively. Manual assessment systematically overestimated biatrial volumes compared to semiautomated measurement (LAVi mean bias 3.4 ± 7.9 mL/m^2^; RAVi mean bias 1 ± 23.3 mL/m^2^). 

**Figure 3:**
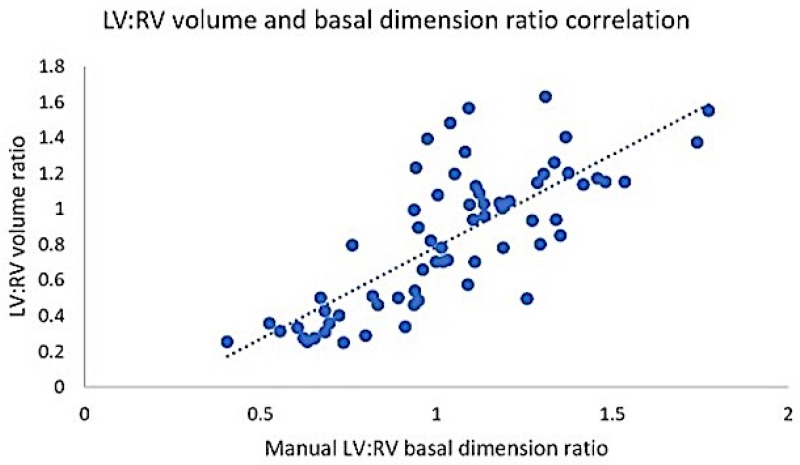
Relationship between semiautomated LV:RV volume ratio and manual basal ventricular dimension ratio. LV: left ventricle; RV: right ventricle.

A strong relationship (Figure 3) was seen between the semiautomated LV:RV volume and manual basal ventricular dimension ratios (r 0.77, 95% CI 0.64–0.85, *p* < 0.001; y = −0.246 + 1.034x). We then tested the ability of the manual LV:RV basal dimension ratio to predict adverse hemodynamics. The LV:RV dimension ratio cut points of <1.03 and ≤0.99 (alternatively expressed as RV:LV basal dimension ratio of >0.97 and ≥1.01) showed excellent performance (*p* < 0.001) for mPAP ≥ 38 mmHg (AUC 0.87) and PVR ≥ 5.3 WU (AUC 0.95), respectively. This technique had a sensitivity and specificity of 84% and 82% for predicting elevated mPAP and 86% and 92% for elevated PVR (Supplementary Table S2). The average analysis time for manual and semiautomated volume evaluation was 3 min and 8 min, respectively. 

## 4. Discussion

Our study demonstrates the feasibility of biatrial and biventricular size quantification in CTEPH on non-ECG-gated CTPA and its usefulness in the identification of significant residual PH post-PTE. 

We chose to express the cardiac chamber alterations using the left to right atrioventricular ratios rather than the conventional right to left ventricular ratios in order to emphasize the smaller and underfilled left heart chambers, as this is often less well recognized compared to the dilatation of the right heart chambers.

It must be emphasized that while both atrial and ventricular size showed a good correlation with various hemodynamic parameters, individual chamber measurements performed less well when compared to volume ratios. Of all the measurements, the ventricular volume ratio had the best correlation with invasive catheter data and also excelled at predicting adverse hemodynamics. A ventricular ratio cut point ≤0.82 had high sensitivity (91% and 97%) and specificity (88% and 85%) for identifying significant elevations of mPAP and PVR, respectively (AUC 0.90 and 0.95).

The CT-derived ventricular volume alterations with increased RV and decreased LV size before surgery can be explained by CTEPH pathophysiology. PVR elevation due to the anatomical obstruction by the thromboembolic material leads to RV dysfunction. With increasing pressure overload, RV contractility increases to preserve systolic function. However, there is a worsening of diastolic function with progressive RV hypertrophy and remodeling. An increase in RA compliance and distensibility augments RV filling and performance to conserve cardiac output and prevent RV failure. LV diastolic impairment is also common in CTEPH and normalizes after successful PTE [14,15]. A decrease in RV stroke volume results in diminished venous return and LA underfilling, contributing to a reduction in LV preload and cardiac output. Furthermore, the right–left ventricular interaction, mediated largely through the interventricular septum with an intact pericardium, is another contributory factor, as leftward septal displacement compresses the LV, reducing its distensibility and preload. An improvement or normalization of septal position after PTE is associated with augmentation of LV filling [16,17].

Another consequence of this interdependence mechanism is the compression and decrease in LA size caused by the enlarging RA. With the growing appreciation that atrial function may be an early and sensitive marker of right heart remodeling and coupling [18], we also explored the effect of atrial size before and after PTE. Our finding of a significant increase in LAVi following successful PTE is congruent with the only other existing study that is based on echocardiography, where the authors showed that LA volume increases significantly after surgery [19]. Whereas Marston et al. [19] found that smaller LAVi by itself had a significant association with lower CO, higher mPAP and PVR, in our population, LAVi in isolation had a good correlation with PVR (*p* < 0.001) but a weaker correlation with mPAP (*p* = 0.003) and no correlation with CI. This could be due to several technical factors. Previous studies [20,21] have shown that LA volumes by TTE are consistently underestimated compared to CT. It is also important to remember that LA volume on echocardiography is measured at end-systole, a technique that has demonstrated the strongest correlation to cardiovascular risk stratification [22], whereas, with non-ECG-gated CTPA, there is no reproducible choice for cardiac cycle phase selection. Notwithstanding these limitations, we found LA:RA ratios had a stronger association with hemodynamic parameters than isolated atrial size measurements. Furthermore, a low LA and high RA volume ratio was associated with higher PVR and mPAP, and lower CO before and after surgery, and hence can be a marker for disease severity. Additionally, the increase in LA and decrease in RA volumes following PTE are significantly correlated with PVR improvement, providing another potential noninvasive marker for postoperative success. 

It must be emphasized that CTPA measurements predicted PVR better than mPAP elevation. This is because the determinants of pulmonary artery pressure include both PVR and RV contractility. While RA size and RV contractility are inversely related [23], the non-ECG-gated nature of atrial measurements may have diminished the strength of this association and contributed to the lower observed predictive ability for mPAP elevation. 

We acknowledge that tricuspid regurgitation can be an important confounder as it is common in CTEPH and contributes to RA enlargement. In our cohort, there was significant improvement in TR post-PTE without any targeted intervention to the tricuspid valve at the time of surgery. This is consistent with previous observations that TR is secondary in nature, specifically due to tricuspid leaflet tethering from RV enlargement and associated annular dilatation [24,25]. Alterations in TV morphology can be fully reversible after PTE, correlating with improvements in right ventricular–pulmonary arterial coupling [26].

The reproducibility of CT cardiac chamber quantification is confirmed by the excellent interclass correlation in our study. Hence, chamber measurements can easily be performed on baseline CTPA acquired for CTEPH diagnosis and on the follow-up CTPA performed for evaluation of residual disease post-PTE. The good agreement between semiautomated and manual measurements for both atrial and ventricular volumes is encouraging. as it allows CT readers to evaluate cardiac chamber size on a routine PACS workstation even if they do not have access to dedicated software. Finally, the manual LV:RV basal dimension ratio may be an expeditious alternative to the automated ventricular volume ratio as it offers comparable prognostic utility. 

Our study has some limitations. The sample size is small, but CTEPH is a relatively uncommon condition, and furthermore, only proximal CTEPH is amenable for surgery, further limiting the cohort size. As CTPA was performed without ECG gating, it was not possible to measure the chamber size at a consistent point in the cardiac cycle. While ECG-gating may potentially improve accuracy, we believe our approach reflects real-world clinical practice, as most institutions perform CTPA without ECG-gating. Manual assessment is operator-dependent, but our quantitative data demonstrated excellent intraclass correlation, and there was overall good agreement between the techniques. We acknowledge that the sophisticated ‘method of discs’ volumetric calculation would have better accounted for atrial geometric distortion (compared to our chosen area–length method) but would have required additional software to compute. We wanted to propose a simple approximation that did not require the latter. 

## 5. Conclusions

The primary findings of our study support the assertion that it is feasible to perform cardiac chamber measurements in CTEPH on non-ECG-gated CT and identify high-risk patients with persistent PH post-PTE. As the management of residual PH is challenging, often requiring individualized treatment regimes, this noninvasive measure by CT can help in the detection of patients at a higher risk of long-term postoperative morbidity and mortality and hence will benefit from closer monitoring and also help in triaging such cases for invasive right heart catheterization. While volumetric atrial assessment on CTPA is achievable as well as reproducible, it was outperformed by ventricular metrics for the prediction of adverse hemodynamics. Furthermore, a simple LV:RV dimension ratio of <1.03 and ≤0.99 (alternatively expressed as an RV:LV ratio of >0.97 and ≥1.01) can identify significant elevations of mPAP and PVR, respectively. We do not propose CTPA-derived cardiac chamber size measurements as a substitute for right heart catheterization but rather as a surrogate noninvasive “biomarker”, particularly as CTPA is a commonly performed investigation for CTEPH diagnosis and has the potential to provide additional data for prognostication. Prospective studies in large-volume CTEPH specialist institutions will be needed to validate whether cardiac chamber measurements on CTPA can be used to guide treatment decisions in combination with other established risk assessment tools. 

## Data Availability

The data presented in this study are available on request from the corresponding author. These data are not publicly available as the study was not registered in the public domain.

## Supplementary Materials

The following supporting information can be downloaded from https://www.mdpi.com/article/10.3390/tomography9050142/s1. Table S1: Clinical and laboratory parameters in the pre- and post-pulmonary endarterectomy groups. Table S2: Manual LV:RV basal dimension ratio to predict adverse hemodynamics.

## Appendix A. Non-ECG-Gated CTPA Methodology for Cardiac Chamber Quantification

The CT cardiac chamber quantification was performed in accordance with the well-established international recommendations in echocardiography [13].

Multiplanar reformats (MPR) of the true axial stacks were used to create the apical four- and two-chamber planes analogous to transthoracic echocardiographic (TTE) views. The long axis reference lines were placed through RV and LV apices and mid tricuspid and mitral valves, and the short axis reference lines were placed parallel and aligned with the tricuspid and mitral annular planes, respectively. The slice height was adjusted cranio-caudally to remove RV and LV outflow tracts from the view. The short axis planes were used for cross-reference to ensure the manually created two- and four-chamber planes transected the appropriate and relevant myocardial segments.

Semiautomated measurements were performed offline using commercially available software (CVI42 version 5.12.1, Circle Cardiovascular Imaging, Calgary, Canada). A combination of a threshold-based segmentation tool and a multi-point tool were used to detect the ventricular endocardial contours. Fine corrections were made with the contour smoothing tool when necessary. The biatrial endocardial borders were delineated using a point-and-click approach before the automated tracking algorithm was applied. The pulmonary veins and atrial appendages were excluded from the analysis. Maximum and minimum volumes were calculated based on the biplane area–length method [13] and indexed to body surface area.

Manual atrial planimetry was performed using the Vitrea Advanced Visualization multimodal platform (Vital Images, Inc.; Minnetonka, MN, USA). A freehand ROI tool was used for tracing the atrial outlines. Pulmonary veins and atrial appendages were excluded. LA volume was estimated by: (0.85 × area 1 (2 chamber) × area 2 (4 chamber)) ÷ shortest LA long axis length (1), and RA volume by: (0.85 × (RA 4 chamber area)^2^) ÷ RA long axis length [13]. A total biventricular length was measured from the four-chamber plane, extending from the widest basal portion of the RV to the LV endocardium. Interventricular septal thickness was assigned to LV, with four-chamber basal LV length given by the difference between total biventricular length and four-chamber basal RV length.

## References

[1] Humbert M., Kovacs G., Hoeper M.M., Badagliacca R., Berger R.M.F., Brida M., Carlsen J., Coats A.J.S., Escribano-Subias P., Ferrari P. (2022). 2022 ESC/ERS Guidelines for the diagnosis and treatment of pulmonary hypertension. Eur. Heart J..

[2] Dardi F., Manes A., Guarino D., Suarez S.M., Loforte A., Rotunno M., Pacini D., Galiè N., Palazzini M. (2022). Long-term outcomes after pulmonary endarterectomy. Ann. Cardiothorac. Surg..

[3] Madani M., Mayer E., Fadel E., Jenkins D.P. (2016). Pulmonary Endarterectomy. Patient Selection, Technical Challenges, and Outcomes. Ann. Am. Thorac. Soc..

[4] Mayer E., Jenkins D., Lindner J., D’armini A., Kloek J., Meyns B., Ilkjaer L.B., Klepetko W., Delcroix M., Lang I. (2011). Surgical management and outcome of patients with chronic thromboembolic pulmonary hypertension: Results from an international prospective registry. J. Thorac. Cardiovasc. Surg..

[5] Miyahara S., Schröder T.A., Wilkens H., Karliova I., Langer F., Kunihara T., Schäfers H.-J. (2021). Long-term Outcomes After Pulmonary Endarterectomy in 499 Patients Over a 20-Year Period. Ann. Thorac. Surg..

[6] Delcroix M., Lang I., Pepke-Zaba J., Jansa P., D’Armini A.M., Snijder R., Bresser P., Torbicki A., Mellemkjaer S., Lewczuk J. (2016). Long-Term Outcome of Patients with Chronic Thromboembolic Pulmonary Hypertension: Results from an International Prospective Registry. Circulation.

[7] Cannon J.E., Su L., Kiely D.G., Page K., Toshner M., Swietlik E., Treacy C., Ponnaberanam A., Condliffe R., Sheares K. (2016). Dynamic Risk Stratification of Patient Long-Term Outcome After Pulmonary Endarterectomy: Results from the United Kingdom National Cohort. Circulation.

[8] Simonneau G., Torbicki A., Dorfmüller P., Kim N. (2017). The pathophysiology of chronic thromboembolic pulmonary hypertension. Eur. Respir. Rev..

[9] Tello K., Dalmer A., Vanderpool R.R., Ghofrani H.A., Naeije R., Roller F., Seeger W., Wiegand M., Gall H., Richter M.J. (2020). Right ventricular function correlates of right atrial strain in pulmonary hypertension: A combined cardiac magnetic resonance and conductance catheter study. Am. J. Physiol.-Heart Circ. Physiol..

[10] Yamasaki Y., Abe K., Kamitani T., Hosokawa K., Kawakubo M., Sagiyama K., Hida T., Matsuura Y., Murayama Y., Funatsu R. (2020). Balloon pulmonary angioplasty improves right atrial reservoir and conduit functions in chronic thromboembolic pulmonary hypertension. Eur. Heart J.-Cardiovasc. Imaging.

[11] Hoit B.D. (2014). Left Atrial Size and Function. J. Am. Coll. Cardiol..

[12] Nagueh S.F., Smiseth O.A., Appleton C.P., Byrd B.F., Dokainish H., Edvardsen T., Flachskampf F.A., Gillebert T.C., Klein A.L., Lancellotti P. (2016). Recommendations for the Evaluation of Left Ventricular Diastolic Function by Echocardiography: An Update from the American Society of Echocardiography and the European Association of Cardiovascular Imaging. Eur. Heart J. Cardiovasc. Imaging.

[13] Lang R.M., Badano L.P., Mor-Avi V., Afilalo J., Armstrong A., Ernande L., Flachskampf F.A., Foster E., Goldstein S.A., Kuznetsova T. (2015). Recommendations for Cardiac Chamber Quantification by Echocardiography in Adults: An Update from the American Society of Echocardiography and the European Association of Cardiovascular Imaging. J. Am. Soc. Echocardiogr..

[14] Mahmud E., Raisinghani A., Hassankhani A., Sadeghi H.M., Strachan G.M., Auger W., DeMaria A.N., Blanchard D.G. (2002). Correlation of left ventricular diastolic filling characteristics with right ventricular overload and pulmonary artery pressure in chronic thromboembolic pulmonary hypertension. J. Am. Coll. Cardiol..

[15] Gurudevan S.V., Malouf P.J., Auger W.R., Waltman T.J., Madani M., Raisinghani A.B., DeMaria A.N., Blanchard D.G. (2007). Abnormal Left Ventricular Diastolic Filling in Chronic Thromboembolic Pulmonary Hypertension. J. Am. Coll. Cardiol..

[16] Lumens J., Blanchard D.G., Arts T., Mahmud E., Delhaas T. (2010). Left ventricular underfilling and not septal bulging dominates abnormal left ventricular filling hemodynamics in chronic thromboembolic pulmonary hypertension. Am. J. Physiol.-Heart Circ. Physiol..

[17] Dittrich H.C., Chow L.C., Nicod P.H. (1989). Early improvement in left ventricular diastolic function after relief of chronic right ventricular pressure overload. Circulation.

[18] Lang I.M., Binder T. (2020). Right atrial strain is a surrogate of coupling in the right heart. Eur. Heart J.-Cardiovasc. Imaging.

[19] Marston N.A., Auger W.R., Madani M.M., Kimura B.J., Strachan G., Raisinghani A.B., DeMaria A.N., Blanchard D.G. (2014). Assessment of left atrial volume before and after pulmonary thromboendarterectomy in chronic thromboembolic pulmonary hypertension. Cardiovasc. Ultrasound.

[20] Koka A.R., Yau J., Van Why C., Cohen I.S., Halpern E.J. (2010). Underestimation of Left Atrial Size Measured with Transthoracic Echocardiography Compared With 3D MDCT. Am. J. Roentgenol..

[21] Koka A.R., Gould S.D., Owen A.N., Halpern E.J. (2012). Left Atrial Volume: Comparison of 2D and 3D Transthoracic Echocardiography with ECG-gated CT Angiography. Acad. Radiol..

[22] Tsang T.S., Abhayaratna W.P., Barnes M.E., Miyasaka Y., Gersh B.J., Bailey K.R., Cha S.S., Seward J.B. (2006). Prediction of Cardiovascular Outcomes with Left Atrial Size. J. Am. Coll. Cardiol..

[23] Fischer L., Benjamin N., Blank N., Egenlauf B., Fischer C., Harutyunova S., Koegler M., Lorenz H.-M., Marra A.M., Nagel C. (2018). Right heart size and function significantly correlate in patients with pulmonary arterial hypertension—A cross-sectional study. Respir. Res..

[24] Sadeghi H.M., Kimura B.J., Raisinghani A., Blanchard D.G., Mahmud E., Fedullo P.F., Jamieson S.W., DeMaria A.N. (2004). Does lowering pulmonary arterial pressure eliminate severe functional tricuspid regurgitation?. J. Am. Coll. Cardiol..

[25] Menzel T., Kramm T., Wagner S., Mohr-Kahaly S., Mayer E., Meyer J. (2002). Improvement of tricuspid regurgitation after pulmonary thromboendarterectomy. Ann. Thorac. Surg..

[26] Frederiksen C.A., Waziri F., Ringgaard S., Mellemkjær S., Clemmensen T.S., Hjortdal V.E., Nielsen S.L., Poulsen S.H. (2021). Reverse remodeling of tricuspid valve morphology and function in chronic thromboembolic pulmonary hypertension patients following pulmonary thromboendarterectomy: A cardiac magnetic resonance imaging and invasive hemodynamic study. BMC Cardiovasc. Disord..

