# Supplementary material for: Computed Tomography Pulmonary Angiography Prediction of Adverse Long-Term Outcomes in Chronic Thromboembolic Pulmonary Hypertension: Correlation with Hemodynamic Measurements Pre- and Post-Pulmonary Endarterectomy
Source: Tomography. 2023 Sep 26;9(5):1787–98. doi: 10.3390/tomography9050142 (PMC10611069; doi:10.3390/tomography9050142)
Supplement: Supplementary file 1 [file tomography-09-00142-s001.zip › Supplementary Table S1.pdf]

Supplementary Table S1:

Clinical and laboratory parameters in the pre and post pulmonary endarterectomy groups

	Pre-op (n=33)	Post-op (n=33)
Age (years)	56 $\pm$ 14	-
Gender (%)	18 M (55%)<br>15 F (45%)	-
BMI (kg/m^2)	31.8 $\pm$ 8.2	
Functional class<br>I/II<br>III/IV	 2 (6%)<br>31 (94%)	 23 (70%)<br>10 (30%)
BNP (ng/L)	255 (84-787)	57 (33-119)
Systolic BP (mmHg)	123 $\pm$ 18	125 $\pm$ 16
HR (bpm)	81 $\pm$ 15	77 $\pm$ 13
eGFR by CKD-EPI (mL/min/1.73 m^2)	74 $\pm$ 13	77 $\pm$ 14
TR grade<br>$\leq$ Mild<br>Moderate<br>Severe	 18 (55%)<br>11 (33%)<br>4 (12%)	 31 (94%)<br>2 (6%)<br>0

BMI: Body Mass Index; BNP: Brain Natriuretic Peptide; BP: Blood Pressure; HR: Heart Rate; eGFR: Glomerular Filtration Rate; TR: Tricuspid Regurgitation.
