# Supplementary material for: Computed Tomography Pulmonary Angiography Prediction of Adverse Long-Term Outcomes in Chronic Thromboembolic Pulmonary Hypertension: Correlation with Hemodynamic Measurements Pre- and Post-Pulmonary Endarterectomy
Source: Tomography. 2023 Sep 26;9(5):1787–98. doi: 10.3390/tomography9050142 (PMC10611069; doi:10.3390/tomography9050142)
Supplement: Supplementary file 1 [file tomography-09-00142-s001.zip › Supplementary Table S2.pdf]

Supplementary Table S2:

Manual LV:RV basal dimension ratio to predict adverse hemodynamics

	Cut point for mPAP ≥ 38 mmHg	AUC of ROC (95% CI)	p-value	Sens.	Spec.
Manual LV:RV basal dimension ratio	≤ 1.03	0.87 (0.76 – 0.94)	<0.001	84%	82%
	Cut point for PVR ≥ 5.3 WU	AUC of ROC (95% CI)	p-value	Sens.	Spec.
Manual LV:RV basal dimension ratio	≤ 0.99	0.95 (0.86 – 0.99)	<0.001	86%	92%
